# Supplementary material for: Identification of errors in draft genome assemblies at single-nucleotide resolution for quality assessment and improvement
Source: Nat Commun. 2023 Oct 17;14:6556. doi: 10.1038/s41467-023-42336-w (PMC10582259; doi:10.1038/s41467-023-42336-w)
Supplement: Supplementary file 1 — Supplementary Information [file 41467_2023_42336_MOESM1_ESM.pdf]

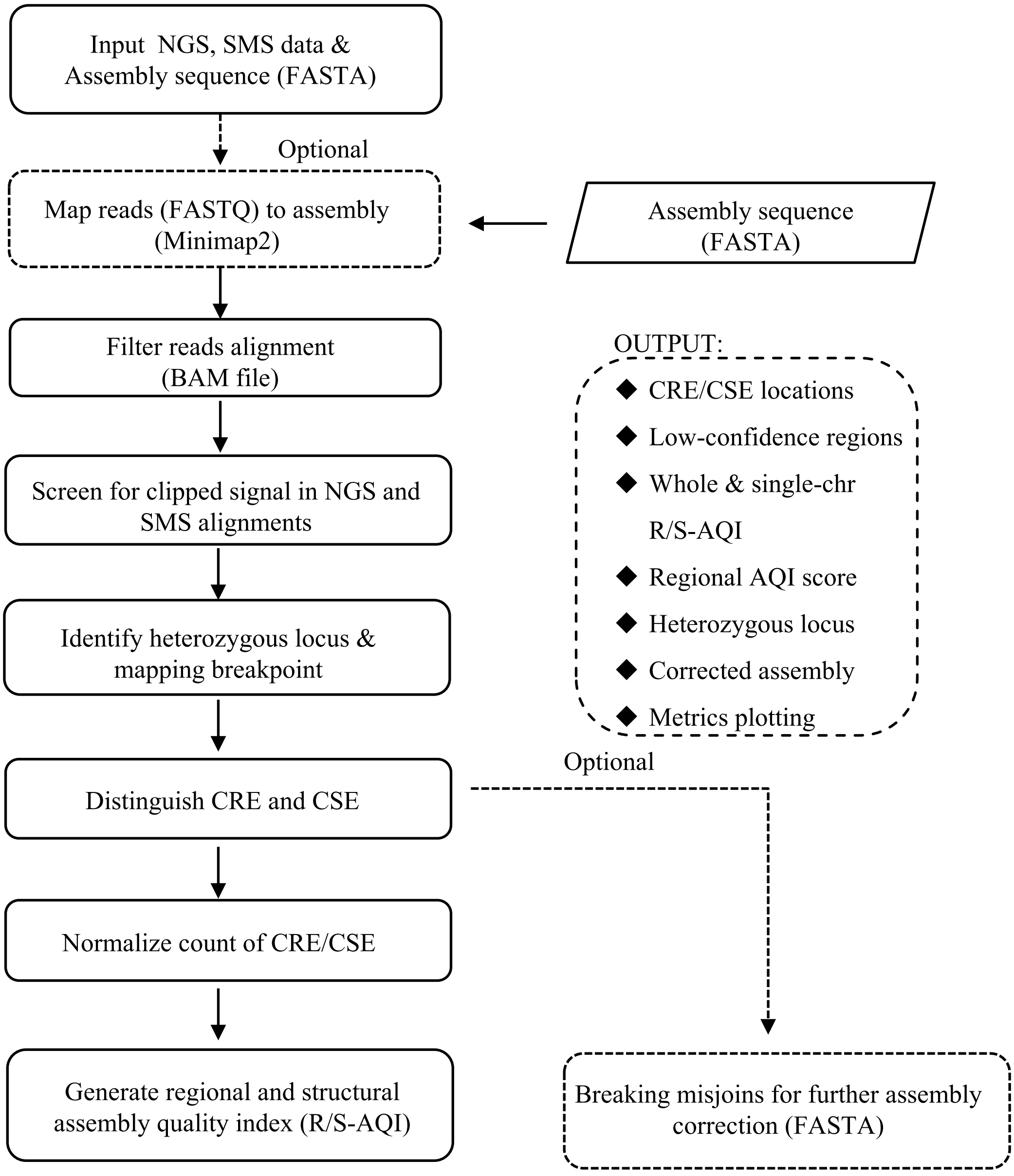

Supplementary Fig. 1 Schematic of the CRAQ algorithm. CRAQ takes the original next-generation sequencing (NGS) short reads and single molecule sequencing (SMS) long reads as input files, and mapped these reads back to the assembly to identify clipped signal. Then, it could report precise breakpoint information, heterozygous variants, regional quality scores based on the alignment information. In addition, CRAQ offers a correction process of splitting these chimeras, which facilitates further refined scaffolding process using Bionano and Hic data.

a

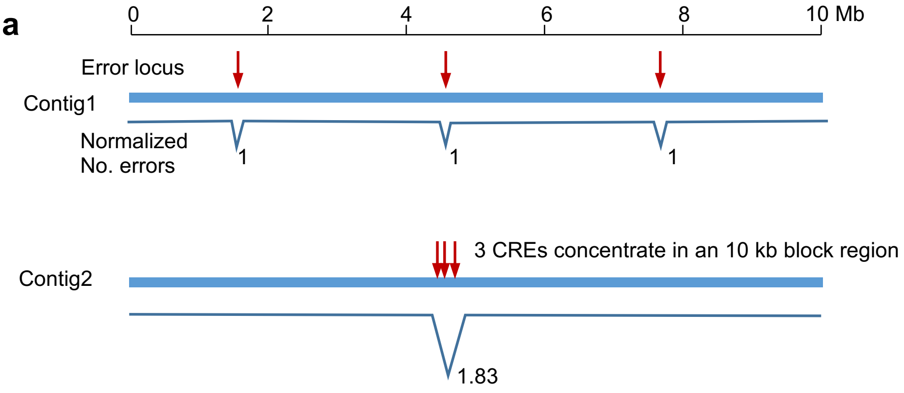

b

	Total real No. errors	Total normalized No. errors	R-AQI score
Contig1	3	3	74
Contig2	3	1.83	83

Supplementary Fig. 2 Theoretical interpretation for error count normalization process. The main purpose of normalization is to avoid some areas with extremely low-quality values to pull down the overall quality score of the assembly. CRAQ outputs both real and normalized number of errors, but the final AQI is calculated based on the normalized number of errors. (a) Assumed 3 CREs were detected on contig1 and contig2, respectively. For contig1: the three CREs spread out on the contig. For contig2, the three CREs located close to each other and within a 10 kb block region. (b) After normalizing, we would get an normalized error number of 3 (equal the real CREs count) for contig1, and 1.83 (1+1/2+1/3) for contig2. The overall AQI was 74 for contig1 and lower than contig2 (AQI = 83).

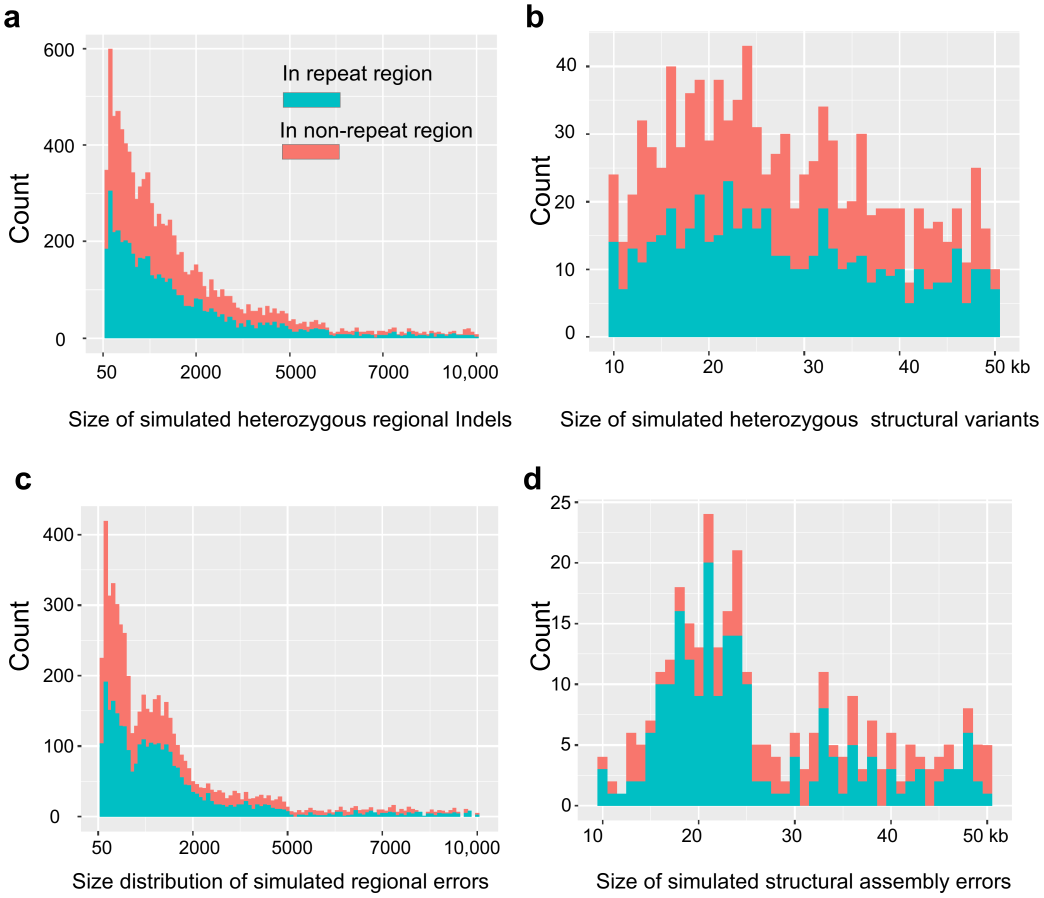

Supplementary Fig. 3 Size distribution of heterozygous variants and simulated errors. Size distribution of simulated heterozygous regional indels (a) and structural variants (b). Size distribution of simulated regional errors (c) and structural errors (d). The peaks at ~1331 bp (in panel c) and ~21 kb (in panel d) indicate the introduce of 1100 small repeat and 100 large fragment repeats from the satellite array of hg38.

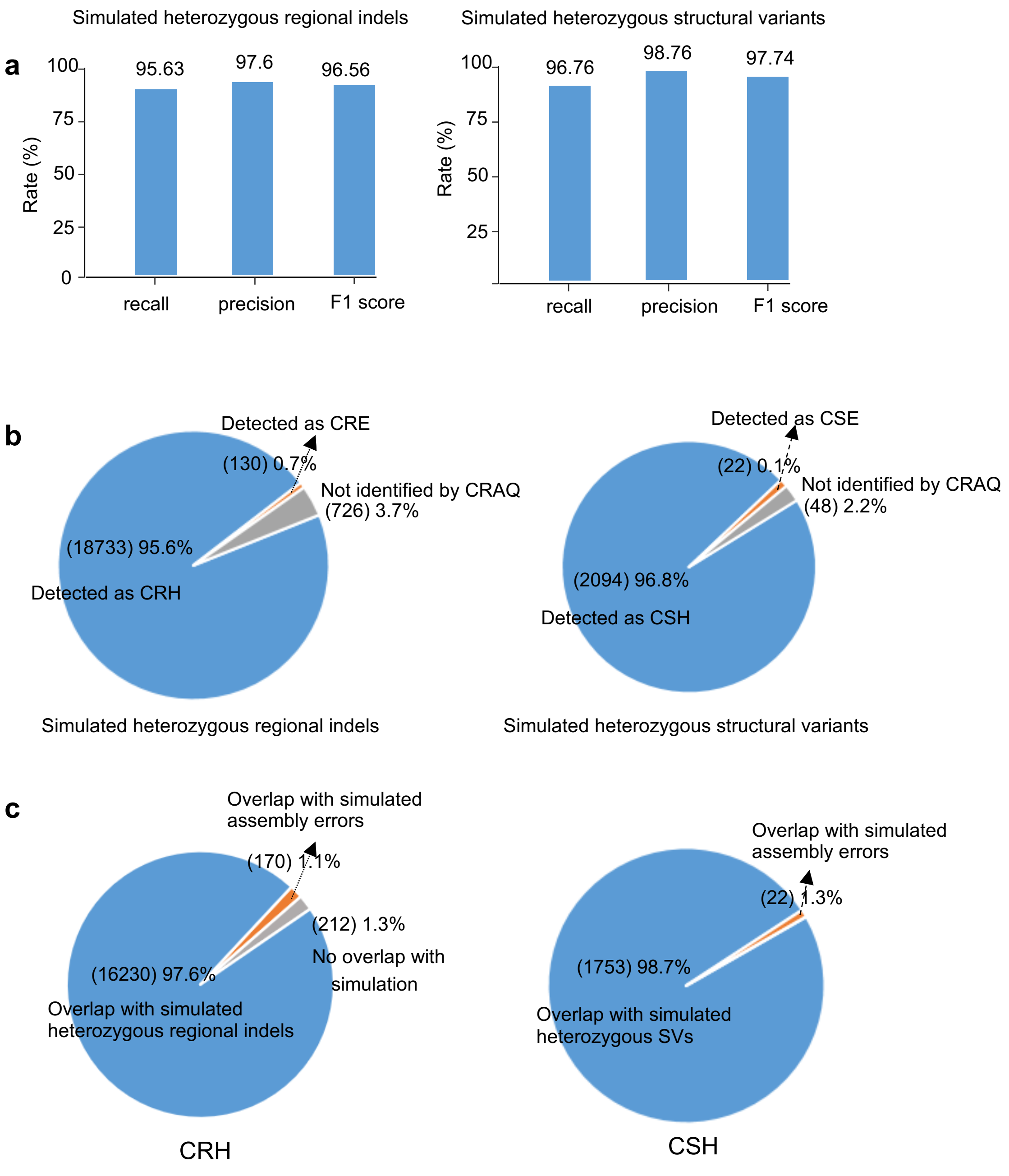

Supplementary Fig. 4 The benchmarking of heterozygous variants detection of CRAQ. (a) The recall, precision and F1 score of simulated heterozygous regional indels (left) and large structural variants (right) by CRAQ. (b) The recall and details for simulated heterozygous indels (left) and structural variants (right). There are very few cases that were detected as assembly errors. (c) The precision and details for CRHs (left) and CSHs (right) of CRAQ's output. There are a few cases that were overlapped with the simulated errors.

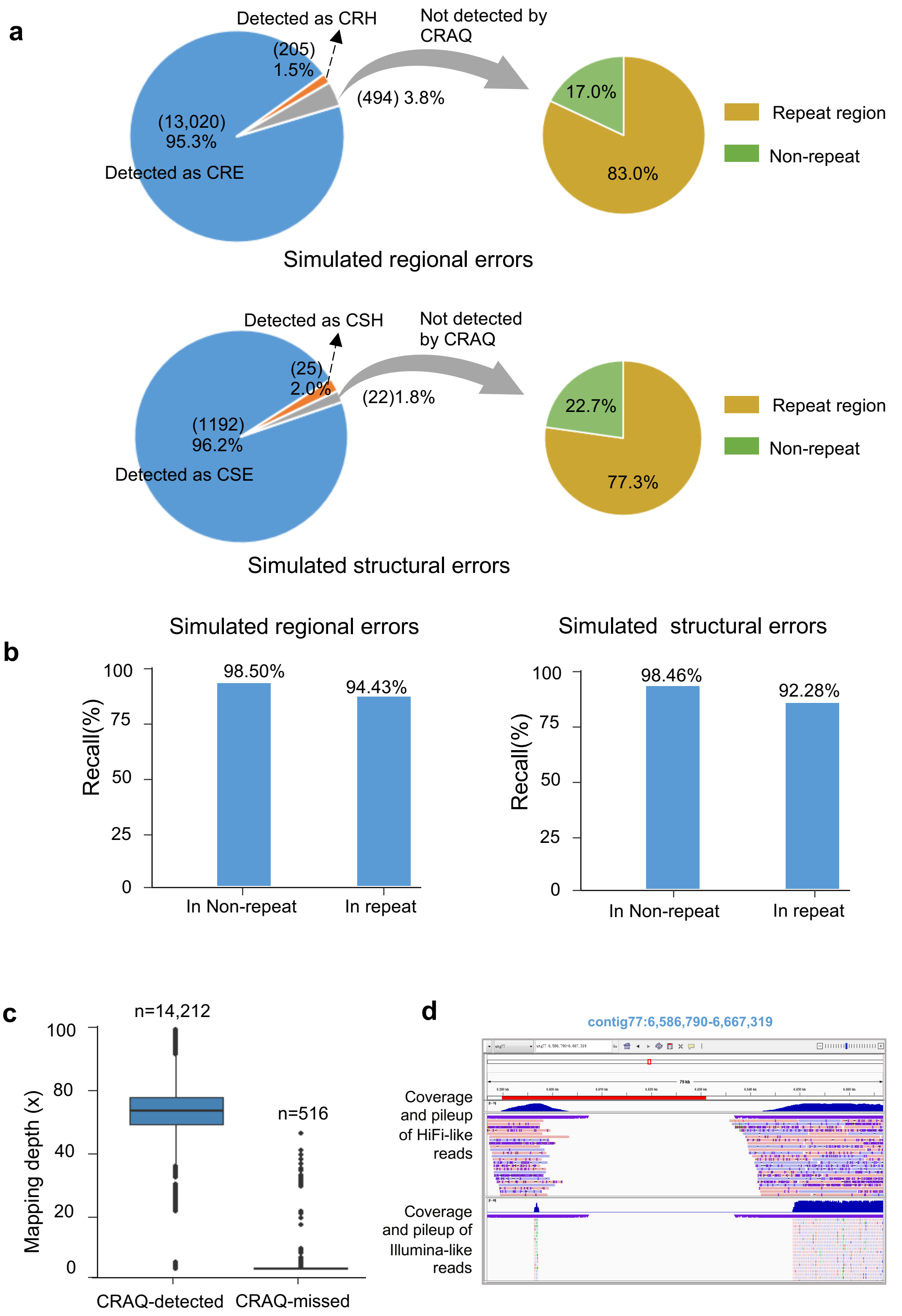

Supplementary Fig. 5 The performance of CRAQ in error detection using simulated data. (a) Pie charts show the numbers of simulated regional/structural assembly errors and CRAQ detected errors or heterozygous variants. 83% of these CRAQ missed regional errors located in the repeats area. 77% of these CRAQ missed structural errors located in the repeats area. (b) The recall rate of simulated errors in repeat and non-repeat regions. (c) The reads mapping depth of CRAQ-detected error and CRAQ-missed error regions. Among the 516 CRAQ-missed errors, 276 were reported as low-confidence regions due to no or limited coverage. (d) An exemplar of the reads mapping status for one of the CRAQ missed errors.

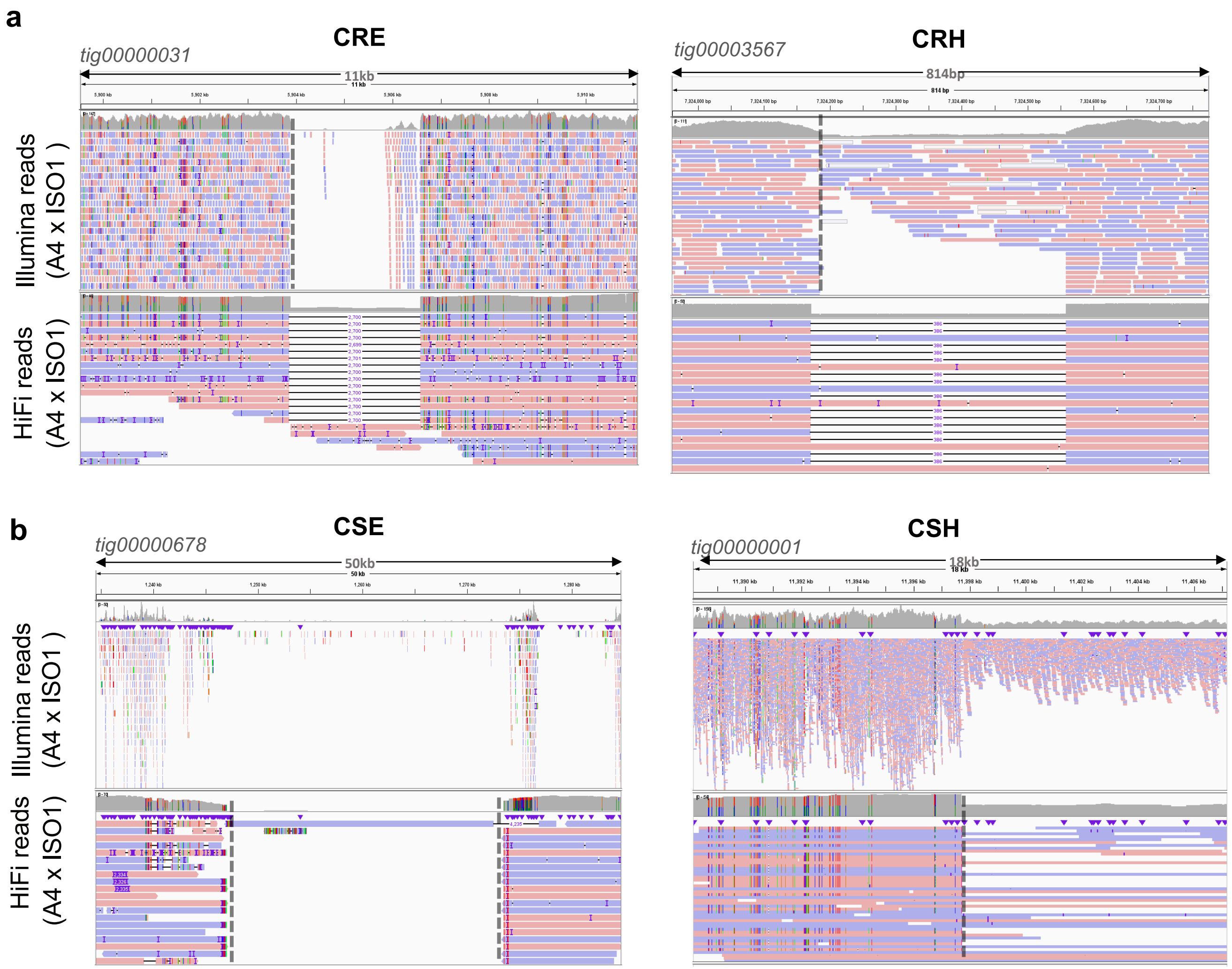

Supplementary Fig. 6 Examples of assembly errors and heterozygous variants identified by CRAQ (a) Reads alignment for an CRE (left) and CRH (right) called by CRAQ for the HiCanu assembly of *D. melanogaster* F1. (b) Reads alignment for an CSE (Left) and CSH (right) called by CRAQ. For CRE/CSE, a substantially high ratio (close to 100%) pileup of NGS clipped reads (for CRE) and SMS clipped reads (for CSE) at the error breakpoint. For CRH/CSH, these regions showed reads pileup with a ratio of error-supporting alignment around 50%. Error breakpoint or heterozygous locus are marked with dashed line.

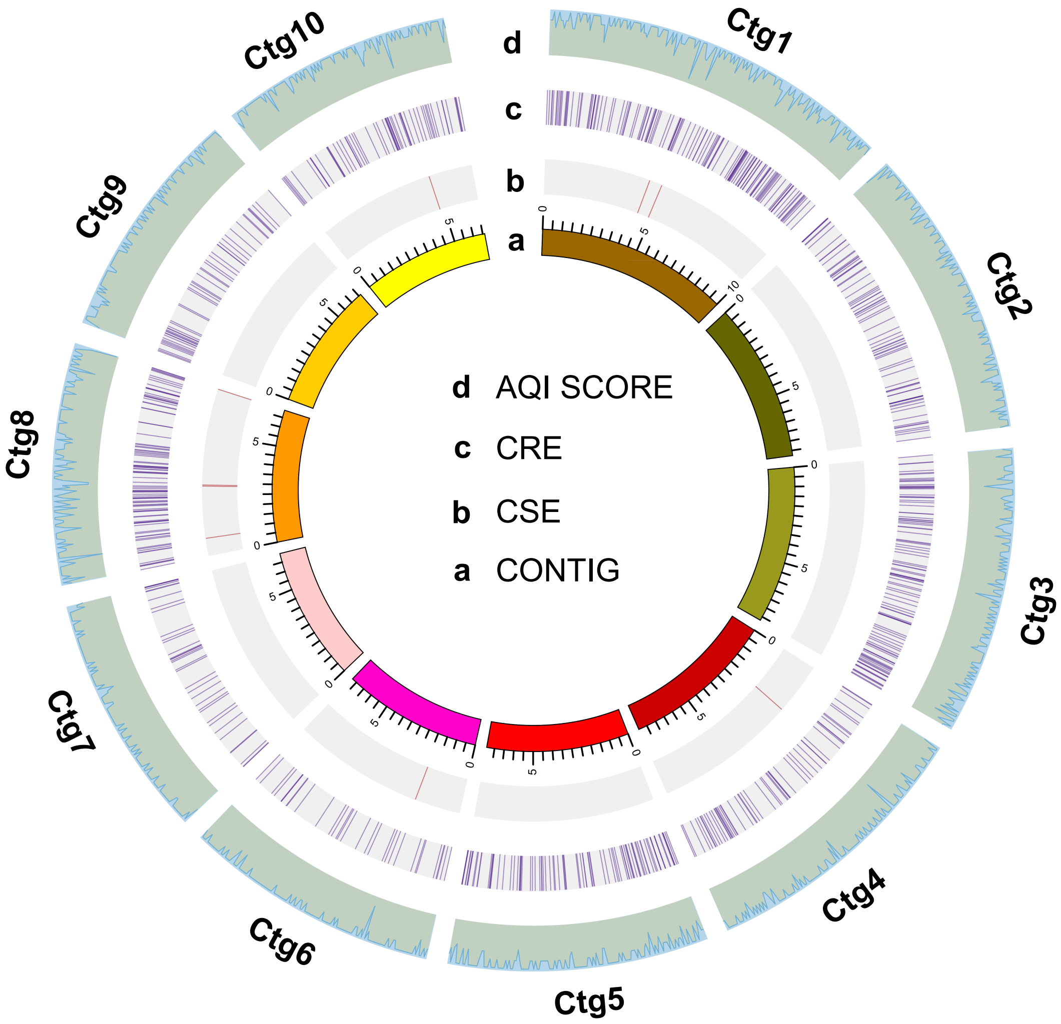

Supplementary Fig. 7 Visualization of CRAQ output for the Canu assembly of *S. pennellii*. Track a displays partial contigs of the Canu assembly. Track b and c present the exact position of CSEs and CREs, respectively, detected by CRAQ. Track d shows regional (50 kb window) comprehensive AQI scores, with the highest values corresponding to error-free assembled regions.

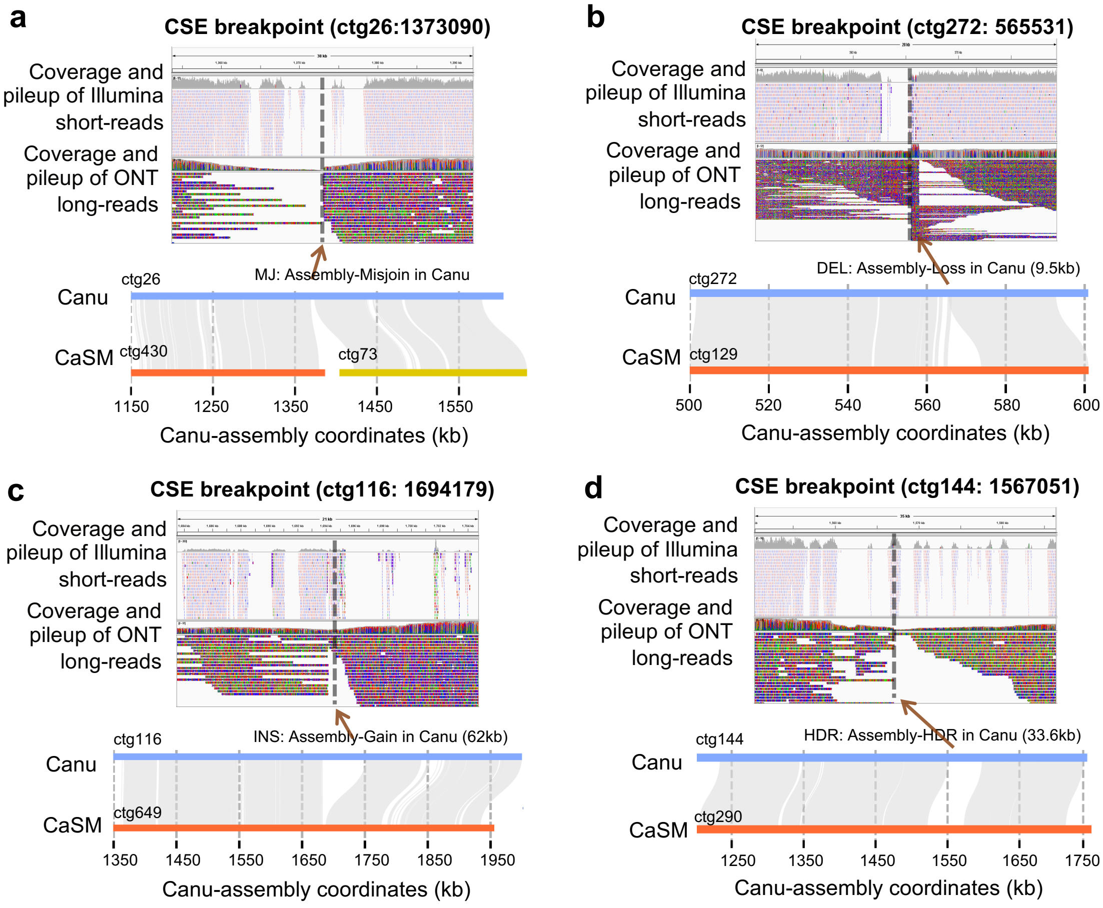

Supplementary Fig. 8 Examples of CSEs and their overlap with SyRI SVs. The upper panel of each sub-figure shows the CSE and the lower panel shows the overlap with different types of errors identified in the Canu assembly of *S. pennellii* reported by SyRI: (a) misjoin events; (b) deletion events; (c) insertion events; and (d) highly divergent regions.

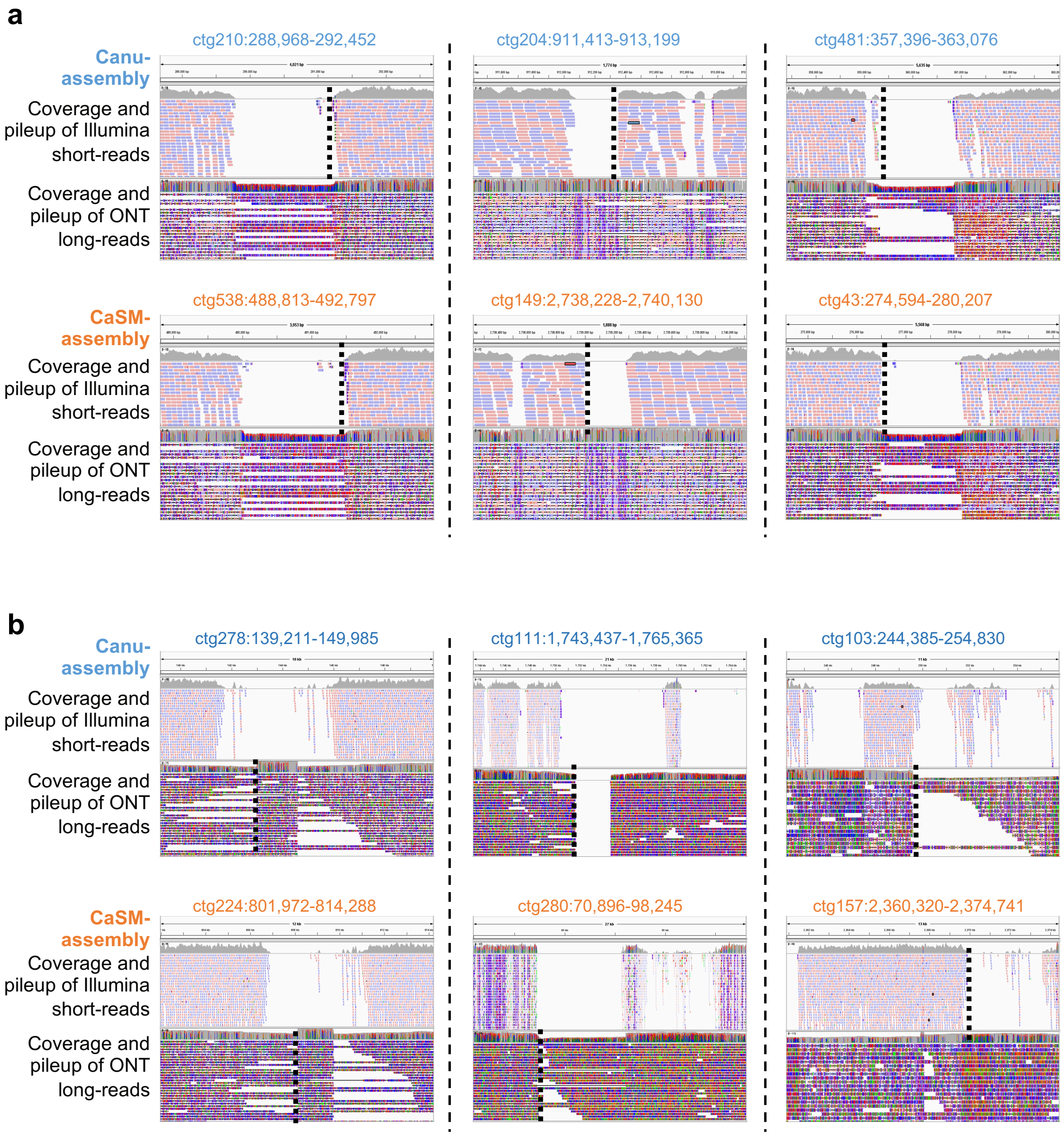

Supplementary Fig. 9 Examples of shared assembly errors between the Canu and CaSM assemblies of *S. pennellii*. The read-mapping status of CRE regions (a) and CSE regions (b) in the Canu assembly (upper panel). The corresponding regions of these CREs/CSEs also exhibited poor mapping statuses in the reference (CaSM) assembly (lower panel).

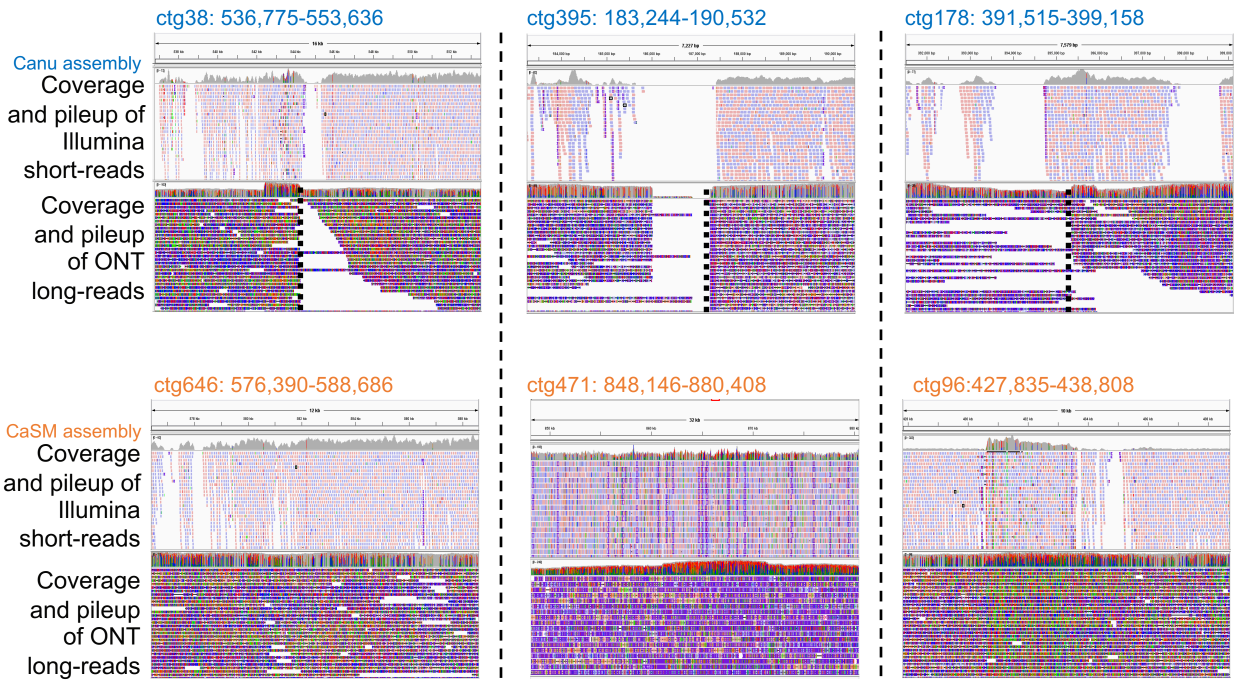

Supplementary Fig. 10 Example alignments of assembly errors specifically detected by CRAQ but not by SyRI. These regions in the Canu assembly (upper panel) exhibited poor read-mapping statuses and were detected as CSEs by CRAQ. The corresponding region in the CaSM assembly exhibited normal coverage (lower panel), but also with noisy mapping status.

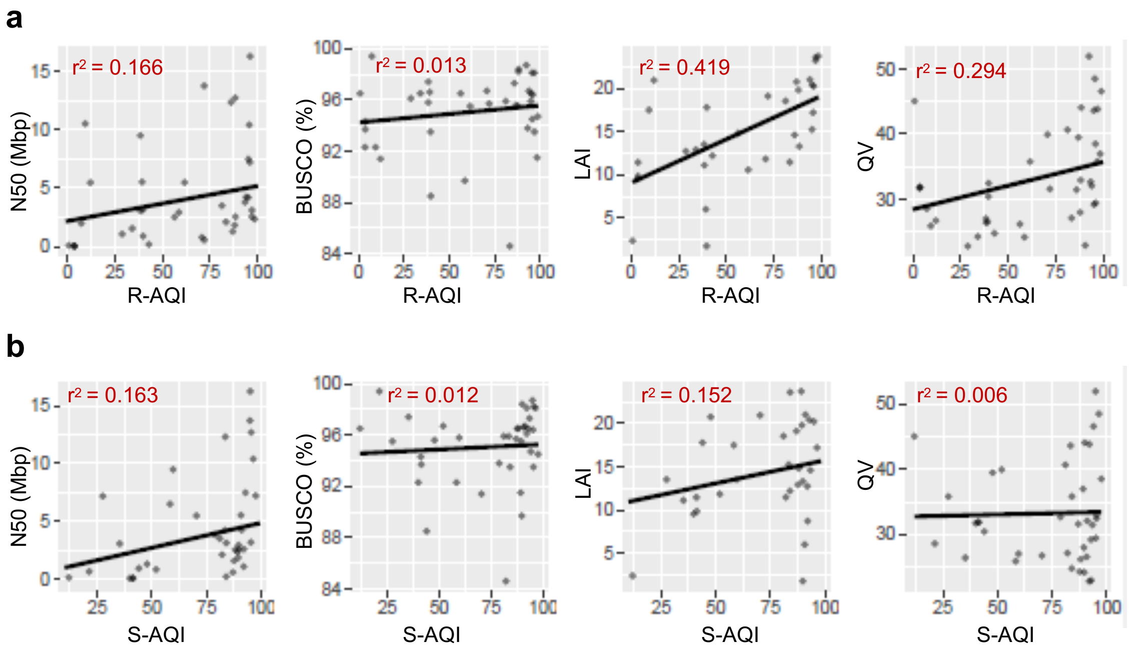

Supplementary Fig. 11 Correlations between R/S-AQI with other benchmarks. Correlation between assembly quality indicator R-AQI (a) S-AQI (b) and other metrics for 40 genomes with variable assembly quality. The specific genomic information is shown in Supplementary Table 1. The coefficient of determination (r^2) are indicated on each plot. Long terminal repeat (LTR) Assembly Index (LAI) could not be calculated for some genomes because LAI is limited to plant genomes with LTR sequences > 5%.

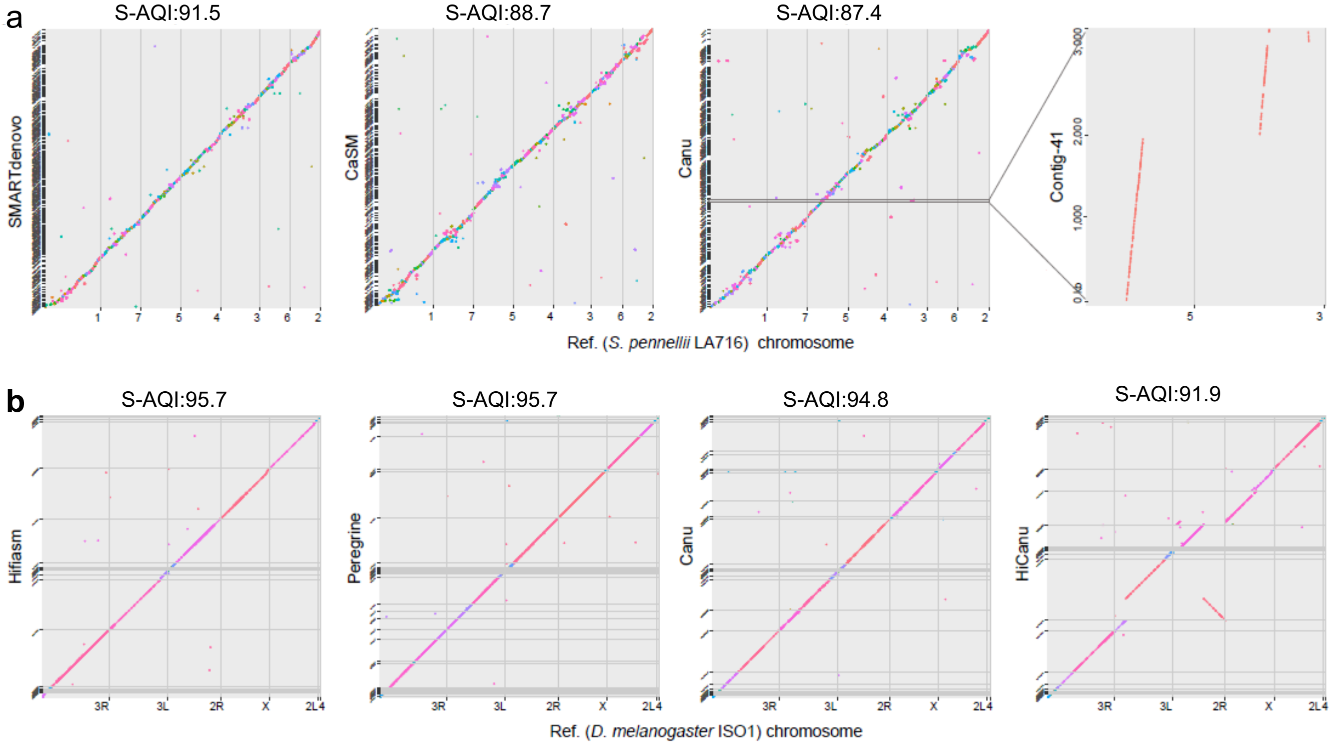

Supplementary Fig. 12 Mummer comparison between assemblies with the reference (a) Dot-plot comparison for the three assemblies of *S. pennellii* LYC1722 against the reference *S. pennellii* LA716. The rightmost panel shows the alignments between contig (contig-15) of CaSM assembly with the reference. (b) Dot-plot comparison for the four primary assemblies of *D. melanogaster* F1 against the reference *D. melanogaster* ISO1. 1-vs-1 unique alignments (>10 kb) from NUCmer (--mum -l 65 -c 200) were used for the dot-plot. Query contig sets were ordered along each reference chromosome by RagTag (https://github.com/malonge/RagTag) and labeled at Y-axis. Each contig is marked with the same color. Short contigs (<50 kbp) were discarded.

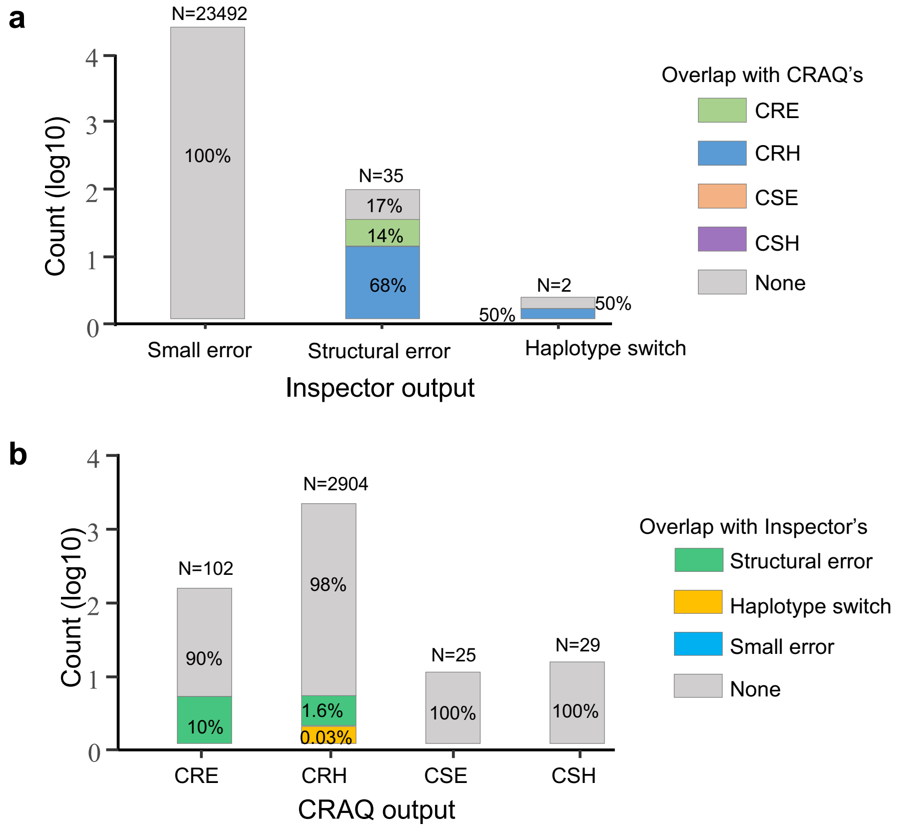

Supplementary Fig. 13 Statistics of the results of Inspector and CRAQ and their overlap. Statistics of the heterozygous/error locus of the HiCanu assembly of *D. melanogaster* F1 detected by Inspector (a) and CRAQ (b). The different colored bars represent the overlaps between Inspector's output with CRAQ's output, with the percentage labeled inside each bar.

Inspector can output three types of assembly errors (Small error; Structural error; Haplotype switch error). All small errors identified by Inspector are indeed tiny SNP/Indel errors, which are not included in CRAQ. 81% of the structural errors (local collapses/expansions) detected by Inspector can be found by CRAQ, but are classified as CRE (14%), CRH (68%). However, all structural errors detected by CRAQ cannot be found by Inspector. All structural heterozygous/error locus are manually checked in IGV.

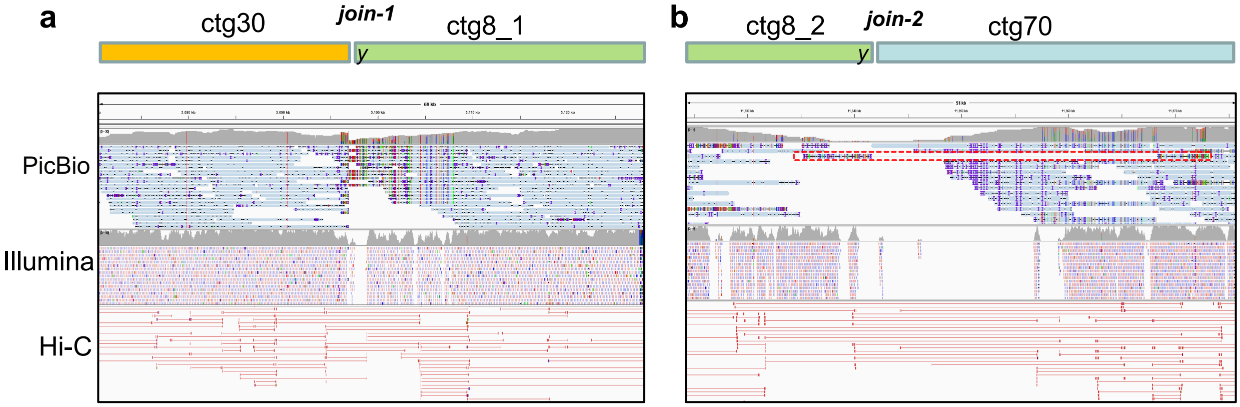

Supplementary Fig. 14 Read alignments in joining regions of the CRAQ-corrected contigs of *A. oxysepala*. (a-b) The read-mapping statuses of new joining regions of ctg8_1 and ctg8_2. Contig8 of *A. oxysepala* (Figure 4a) was identified as a misjoin and split into ctg8_1 and ctg8_2 by CRAQ at the CSE breakpoint (position y). After Hi-C scaffolding, ctg8_1 and ctg8_2 were newly connected with ctg30 and ctg70, respectively. When reads were mapped back to the corrected scaffold, both joined regions (***join-1*** and ***join-2***) were spanned by PacBio long reads, and thus no longer presented as CSE features. Notably, only one PacBio read (outlined in a red dashed line) went across ***join-2***. The mapping statuses of PacBio long reads, Illumina short reads, and Hi-C paired-end reads are shown in the lower panel; Hi-C read pairs exceeding 50 kb in inner distance were removed.

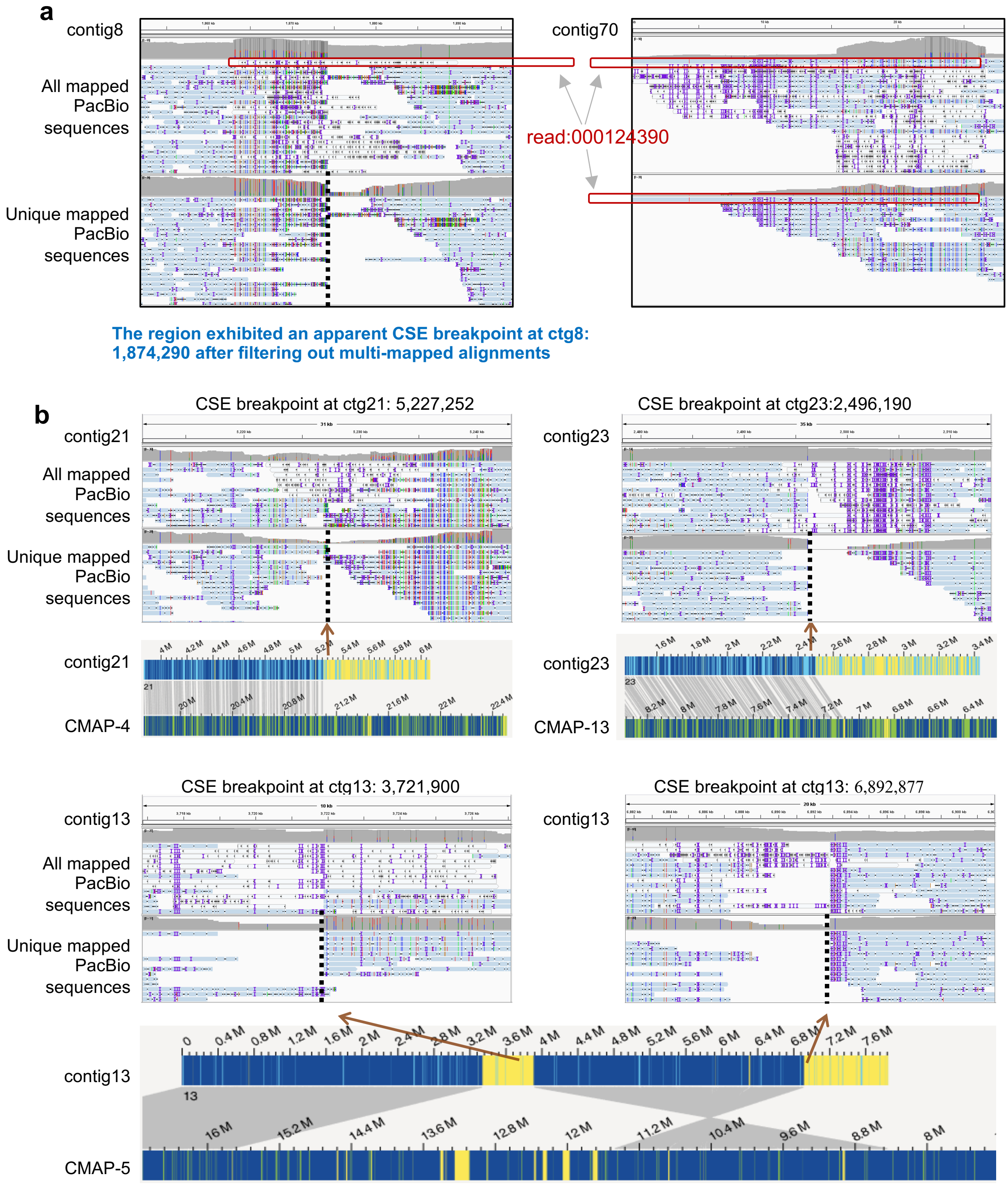

Supplementary Fig. 15 Overview of alignments before and after filtering out multi-mapped reads. (a) The chimeric contig8 of *A. oxysepala* shown in Figure 4A is shown here for reference. When mapping all PacBio reads to the contig, the misjoined error region was spanned by reads. In this case, CRAQ could not detect the error. In fact, only multi-mapped reads crossed the region. These multi-mapped reads (outlined in red) were mapped to two regions (ctg8:1,863,640-1,889,733 and ctg70:1-26,015), indicating the sequence similarity. After filtering out multi-alignments, an apparent CSE breakpoint was found at that region. (b) Read-mapping statuses of other misjoined errors (upper and middle panels). Like contig8, only when filtering out multi-alignments could CSE breakpoints be identified in these misjunction regions. Misjoin events were validated by Bionano optical mapping (lower panel).

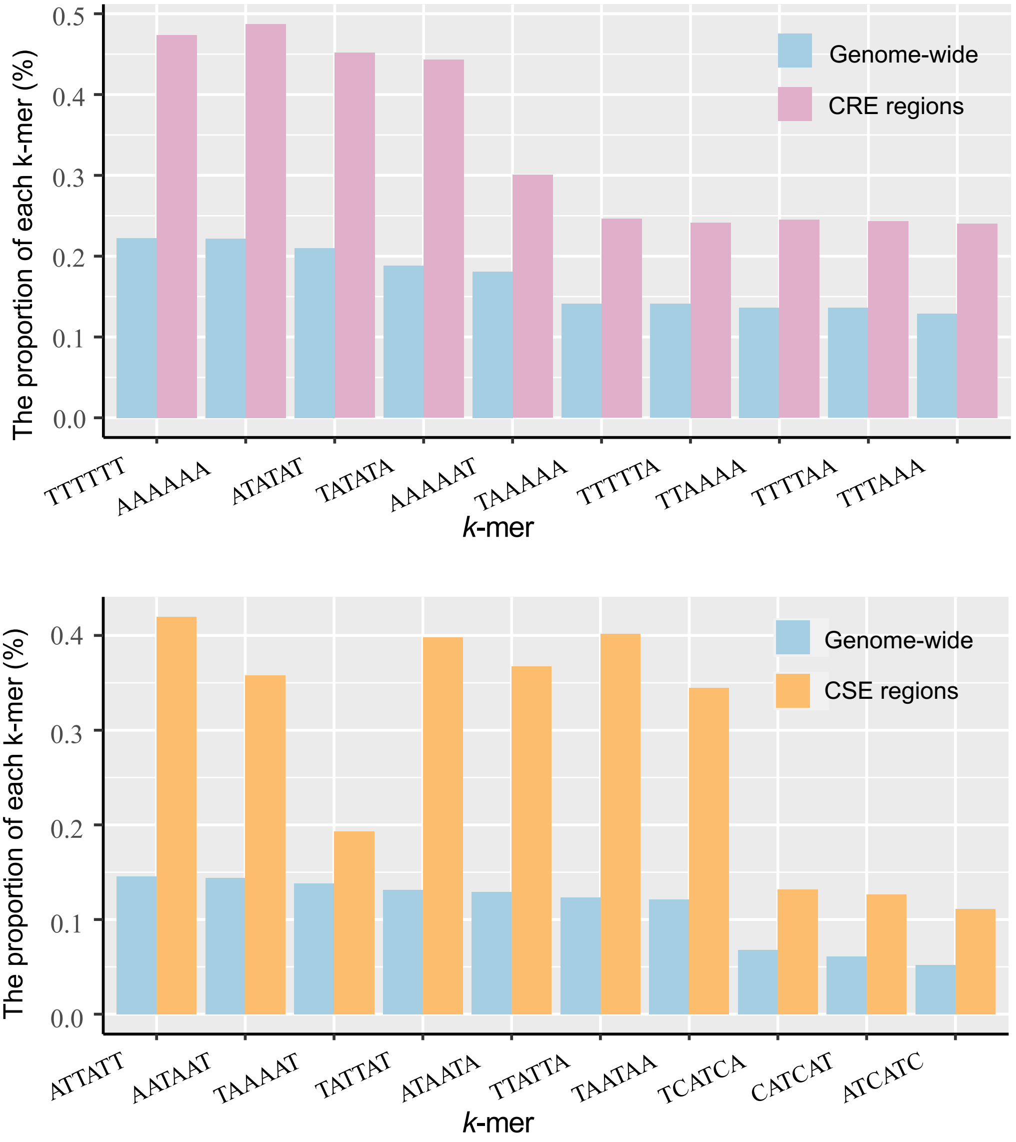

Supplementary Fig. 16 Distribution of sequence contexts within the CRE and CSE regions. 6-mer genomic context features of CRE and CSE regions, with 200 bp flanking each CRE/CSE breakpoint. The sequences of the whole genome were used as the background. The top ten 6-mers enriched in the CRE/CSE regions are noted.
