# Supplementary material for: Identification of errors in draft genome assemblies at single-nucleotide resolution for quality assessment and improvement
Source: Nat Commun. 2023 Oct 17;14:6556. doi: 10.1038/s41467-023-42336-w (PMC10582259; doi:10.1038/s41467-023-42336-w)
Supplement: Supplementary file 3 — Reporting Summary [file 41467_2023_42336_MOESM3_ESM.pdf]

nature portfolio

Corresponding author(s): Yuannian Jiao

Last updated by author(s): Sep 27, 2023

Reporting Summary

Nature Portfolio wishes to improve the reproducibility of the work that we publish. This form provides structure for consistency and transparency in reporting. For further information on Nature Portfolio policies, see our Editorial Policies and the Editorial Policy Checklist.

Statistics

For all statistical analyses, confirm that the following items are present in the figure legend, table legend, main text, or Methods section.

n/a	Confirmed	
[ ]	[x]	The exact sample size (*n*) for each experimental group/condition, given as a discrete number and unit of measurement
[x]	[ ]	A statement on whether measurements were taken from distinct samples or whether the same sample was measured repeatedly
[x]	[ ]	The statistical test(s) used AND whether they are one- or two-sided<br>*Only common tests should be described solely by name; describe more complex techniques in the Methods section.*
[x]	[ ]	A description of all covariates tested
[x]	[ ]	A description of any assumptions or corrections, such as tests of normality and adjustment for multiple comparisons
[ ]	[x]	A full description of the statistical parameters including central tendency (e.g. means) or other basic estimates (e.g. regression coefficient) AND variation (e.g. standard deviation) or associated estimates of uncertainty (e.g. confidence intervals)
[x]	[ ]	For null hypothesis testing, the test statistic (e.g. *F*, *t*, *r*) with confidence intervals, effect sizes, degrees of freedom and *P* value noted<br>*Give P values as exact values whenever suitable.*
[x]	[ ]	For Bayesian analysis, information on the choice of priors and Markov chain Monte Carlo settings
[x]	[ ]	For hierarchical and complex designs, identification of the appropriate level for tests and full reporting of outcomes
[x]	[ ]	Estimates of effect sizes (e.g. Cohen's *d*, Pearson's *r*), indicating how they were calculated

Our web collection on statistics for biologists contains articles on many of the points above.

Software and code

Policy information about availability of computer code

Data collection

The human reference assembly (GRCh38) is download at https://www.ncbi.nlm.nih.gov/datasets/genome/GCF_000001405.26/ . Repeat regions were randomly selected from the repeat database of hg38 (hgdownload.soe.ucsc.edu/hubs/RepeatBrowser2020/hg38/hg38_2020_rmsk.bed). The generated hg38_sim1 and hg38_sim2 genome sequences have been deposited in the zenodo database under accession code https://doi.org/10.5281/zenodo.8383281. The other genome assembly data used in this study were downloaded from their corresponding databases, and the links are listed in Supplemental Data 1.

Data analysis

The used softwares and custom codes were provided as following:
Genome alignment tools:
Minimap2 v2.18 (https://github.com/lh3/minimap2),
SAMTools v1.9 (https://github.com/samtools/samtools)
(./minimap2 -a genome.fa HiFi.fa -x map-hifi > out.sam && samtools view -F 1796 -q20 out.sam -b -h -o out.bam)

Genome assembly:
Hifiasm v0.16.1-r375 (https://github.com/chhylp123/hifiasm)
(./hifiasm -o result -t40 -f0 SMS.fa.gz 2> result.log && awk '/^S/{print ">"$2;print $3}' result.bp.p_ctg.gfa > result.p_ctg.fa)

RefAligner in Bionano Solve Pipeline v3.3 (https://bionanogenomics.com/support/software-downloads/)
(python ~/software/Solve3.3_10252018/Pipeline/10252018/runCharacterize.py -t ~/software/Solve3.3_10252018/RefAligner/7915.7989rel/RefAligner -q query.cmap -r ref.cmap -p ~/software/Solve3.3_10252018/Pipeline/10252018/ -a ~/software/Solve3.3_10252018/RefAligner/7915.7989rel/optArguments_nonhaplotype_noES_noCut_saphyr.xml -n 20 >alignmentstatistics.out)

```
Juicer v1.7.6, Juicebox v1.8.8.8 and 3D-DNA v180114. (https://github.com/aidenlab/juicer)
(~/myJuicerDir/juicer/misc/generate_site_positions.py  MboI draft draft.fa &
 ./scripts/juicer.sh -s MboI -g gennome -z references/genome.fa -y restriction_sites/genome_MboI.txt -p genome.sizes -D /data/myJuicerDir/
genome -t 20
/data/software/myJuicerDir/3d-dna/run-asm-pipeline.sh ../../references/genome.fa ../merged_nodups.txt)

Genome quality assessments:
BUSCO v5.4.6  (https://anaconda.org/bioconda/busco)
 (run_BUSCO.py -i assembly.fasta -o assembly_embryophyta_odb10.busco.out -l embryophyta_odb10 -m genome -c 20)

LTR_retriever v2.9.0 (https://github.com/oushujun/LTR_retriever/releases)
(ltrharvest -db genome.fa -index genome.fa  -mintsd 4 - maxtsd 6 -motif TGCA -motifmis 1 -similar 85 -vic 10 -seed 20 &&
LTR_FINDER -seq genome.fa -threads 10  -size 1000000 -time 300 -D 15000 -d 1000 -L 7000 -l 100 -p 20 -C -M 0.85
cat genome.fa.harvest.scn genome.fa.finder.scn > genome.fa.rawLTR.scn
LTR_retriever -genome genome.fa -inharvest genome.fa.rawLTR.scn -threads 10)

Merqury v1.3 (https://github.com/marbl/merqury)
(meryl k=21 count output read1.meryl read2.fastq.gz
meryl union-sum output genome.meryl read*.meryl
merqury.sh read-db.meryl asm1.fasta out_prefix)

Inspector version1.2 (https://github.com/ChongLab/Inspector)
inspector.py -c contig.fa -r ccsreads.1.fastq ccsreads.2.fastq -o inspector_out --datatype hifi

CRAQ1.0.8 (https://github.com/JiaoLaboratory/CRAQ)
craq  -g  assembly.fa -sms SMS.fa.gz -ngs NGS_R1.fa.gz,NGS_R2.fa.gz -x map-hifi

Genome comparation:
SyRI v1.5 (https://github.com/schneebergerlab/syri)
(syri -r  refgenome -q querygenome  -d delta -nc 20 )
Nucmer v4.0.0beta2 (https://github.com/mummer4/mummer)
(nucmer ref.fa query.fa --mum -l 65 -c 200)

Genome assessment metrics plotting: pycircos (python 3.7later) (https://github.com/JiaoLaboratory/CRAQ)
(CRAQcircos.py --genome_size genome.size --genome_error_loc CRE_CSE.loc  --genome_score regional.AQI.bdg)
```

For manuscripts utilizing custom algorithms or software that are central to the research but not yet described in published literature, software must be made available to editors and reviewers. We strongly encourage code deposition in a community repository (e.g. GitHub). See the Nature Portfolio guidelines for submitting code & software for further information.

Data

Policy information about availability of data

All manuscripts must include a data availability statement. This statement should provide the following information, where applicable:

- Accession codes, unique identifiers, or web links for publicly available datasets
- A description of any restrictions on data availability
- For clinical datasets or third party data, please ensure that the statement adheres to our policy

The human reference assembly (GRCh38) is download at https://www.ncbi.nlm.nih.gov/datasets/genome/GCF_000001405.26/ . Repeat regions were randomly selected from the repeat database of hg38 (hgdownload.soe.ucsc.edu/hubs/RepeatBrowser2020/hg38/hg38_2020_rmsk.bed). The generated hg38_sim1 and hg38_sim2 genome sequences have been deposited in the zenodo database under accession code https://doi.org/10.5281/zenodo.8383281. The other genome assembly data used in this study were downloaded from their corresponding databases, and the links are listed in Supplemental Data 1.

Human research participants

Policy information about studies involving human research participants and Sex and Gender in Research.

Reporting on sex and gender	N/A
Population characteristics	N/A
Recruitment	N/A
Ethics oversight	N/A

Note that full information on the approval of the study protocol must also be provided in the manuscript.

Field-specific reporting

Please select the one below that is the best fit for your research. If you are not sure, read the appropriate sections before making your selection.

☒ Life sciences ☐ Behavioural & social sciences ☐ Ecological, evolutionary & environmental sciences

For a reference copy of the document with all sections, see nature.com/documents/nr-reporting-summary-flat.pdf

Life sciences study design

All studies must disclose on these points even when the disclosure is negative.

Sample size	40 genomes assembled with different sequencing platforms (including ONT, Pacbio and Illumina sequencing technology) and different assembly tools were selected for evaluation in our project. The correlation between R-AQI/SAQI with other indicators including BUSCO values, LAI score, QV values, contigN50 length were denoted by R square.
Data exclusions	For hg38 genomic sequences, contigs with length less than 500k were excluded.
Replication	N/A
Randomization	Genomic loci used to simulate heterozygous variants and assembly errors were randomly selected across the hg38 contigs. Repeat errors were randomly selected from the repeat database (hgdownload.soe.ucsc.edu/hubs/RepeatBrowser2020/hg38reps/) of hg38 and occupied ~10% of the satellite array in hg38.
Blinding	N/A

Reporting for specific materials, systems and methods

We require information from authors about some types of materials, experimental systems and methods used in many studies. Here, indicate whether each material, system or method listed is relevant to your study. If you are not sure if a list item applies to your research, read the appropriate section before selecting a response.

Materials & experimental systems

n/a	Involved in the study
☒	☐ Antibodies
☒	☐ Eukaryotic cell lines
☒	☐ Palaeontology and archaeology
☒	☐ Animals and other organisms
☒	☐ Clinical data
☒	☐ Dual use research of concern

Methods

n/a	Involved in the study
☒	☐ ChIP-seq
☒	☐ Flow cytometry
☒	☐ MRI-based neuroimaging
