# Supplementary material for: Identification of errors in draft genome assemblies at single-nucleotide resolution for quality assessment and improvement
Source: Nat Commun. 2023 Oct 17;14:6556. doi: 10.1038/s41467-023-42336-w (PMC10582259; doi:10.1038/s41467-023-42336-w)
Supplement: Supplementary file 4 — Description of Additional Supplementary Files [file 41467_2023_42336_MOESM4_ESM.pdf]

Description of Additional Supplementary Files

Supplementary Data 1
Description: Public datasets used for assembly quality assessment

Supplementary Data 2
Description: Introduced heterozygous variants and errors in the simulated hg38 assemblies

Supplementary Data 3
Description: Comparison of CRAQ output and simulated errors in the simulated hg38_sim2

Supplementary Data 4
Description: Simulated errors missed by CRAQ (false negatives)

Supplementary Data 5
Description: Potential error locus in the HiCanu assembly of D. melanogaster hybrid F1

Supplementary Data 6
Description: CREs and CSEs identified in the Canu assembly of S. pennellii

Supplementary Data 7
Description: SyRI SV statistics for the Canu assembly of S. pennellii

Supplementary Data 8
Description: Statistics for CSE breakages and overlaps with BioNano conflicts in A. oxysepala

Supplementary Data 9
Description: Bionano conflicts in A. oxysepala and overlaps with CSEs

Supplementary Data 10
Description: Comparison of quality evaluation for A. oxysepala before and after CRAQ correction
